# Identification and Characterization of Calcium Sparks in Cardiomyocytes Derived from Human Induced Pluripotent Stem Cells

**DOI:** 10.1371/journal.pone.0055266

**Published:** 2013-02-07

**Authors:** Guang Qin Zhang, Heming Wei, Jun Lu, Philip Wong, Winston Shim

**Affiliations:** 1 Research and Development Unit, National Heart Centre Singapore, Singapore, Republic of Singapore; 2 Department of Clinical Pharmacy, China Pharmaceutical University, Nanjing, China; 3 Cardiovascular and Metabolic Disorders Program, DUKE-NUS Graduate Medical School Singapore, Singapore, Republic of Singapore; Indiana University School of Medicine, United States of America

## Abstract

**Introduction:**

Ca^2+^ spark constitutes the elementary units of cardiac excitation-contraction (E-C) coupling in mature cardiomyocytes. Human induced pluripotent stem cell (hiPSC)-derived cardiomyocytes are known to have electrophysiological properties similar to mature adult cardiomyocytes. However, it is unclear if they share similar calcium handling property. We hypothesized that Ca^2+^ sparks in human induced pluripotent stem cell (hiPSCs)-derived cardiomyocytes (hiPSC-CMs) may display unique structural and functional properties than mature adult cardiomyocytes.

**Methods and results:**

Ca^2+^ sparks in hiPSC-CMs were recorded with Ca^2+^ imaging assay with confocal laser scanning microscopy. Those sparks were stochastic with a tendency of repetitive occurrence at the same site. Nevertheless, the spatial-temporal properties of Ca^2+^ spark were analogous to that of adult CMs. Inhibition of L-type Ca^2+^ channels by nifedipine caused a 61% reduction in calcium spark frequency without affecting amplitude of those sparks and magnitude of caffeine releasable sarcoplasmic reticulum (SR) Ca^2+^ content. In contrast, high extracellular Ca^2+^ and ryanodine increased the frequency, full width at half maximum (FWHM) and full duration at half maximum (FDHM) of spontaneous Ca^2+^ sparks.

**Conclusions:**

For the first time**,** spontaneous Ca^2+^ sparks were detected in hiPSC-CMs. The Ca^2+^ sparks are predominately triggered by L-type Ca^2+^ channels mediated Ca^2+^ influx, which is comparable to sparks detected in adult ventricular myocytes in which cardiac E-C coupling was governed by a Ca^2+^-induced Ca^2+^ release (CICR) mechanism. However, focal repetitive sparks originated from the same intracellular organelle could reflect an immature status of the hiPSC-CMs.

## Introduction

Excitation-contraction (E-C) coupling in the adult mammalian heart is governed by the Ca^2+^-induced Ca^2+^ release (CICR) mechanism. The process involves entry of Ca^2^ through L-type Ca^2+^ channel that activates the ryanodine receptors (RyRs)-mediated Ca^2+^ release from sarcoplasmic reticulum (SR) and resulting in intracellular Ca^2+^ transients [1]. Ca^2+^ sparks, a local and transient Ca^2+^ release originating from a single RyR or a cluster of RyRs, constitute the elementary events of cardiac E-C coupling [2]. Whole cell Ca^2+^ transients are believed to represent the recruitment and summation of many Ca^2+^ sparks after an increase in opened L-type Ca^2+^ channels [3]. RyR Ca^2+^ release channel is tightly linked to the gating of L-type Ca^2+^ channel and plays a key role in the intracellular Ca^2+^-handling in cardiac myocytes [4]. Such property of Ca^2+^ sparks may reflect the organizational maturity of RyRs in the cardiomyocytes [5].

Human induced pluripotent stem cells (hiPSCs) can be generated by somatic reprogramming of fibroblasts with a set of transcription factors and differentiated into multiple cell lineages, including cardiomyocytes [6]. In contrast to human embryonic stem cells (hESCs), hiPSCs are capable of giving rise to a renewable source of cardiomyocytes (CMs) from individuals. These hiPSC-derived cardiomyocytes (hiPSC-CMs) offer immensely valuable tool in personalized pharmaceutical evaluation of therapeutic agents such as anti-arrhythmics and provide a tenable means for cardiomyocytes replacement therapy. Therefore, it is important to understand the functional characteristics of such hiPSC-CMs, especially in the cardiac excitation-contraction (E-C) coupling. Whole-cell Ca^2+^ transients in hiPSC-CMs are dependent on the CICR mechanism in which the key calcium-handling proteins include RyR2, SERCA2a and L-type Ca^2+^ channels [7], [8]. Early studies with mouse ESC-CMs have suggested that developmental stage of cardiac cell differentiation are related to the triggering of Ca^2+^ sparks and different properties of Ca^2+^ sparks [9]. However, the characteristics of Ca^2+^ sparks in hiPSC-CMs remain unclear.

We hypothesized that the Ca^2+^ sparks in hiPSC-derived cardiomyocytes play instrumental roles in their structural and functional maturity. To better understand regulation of E–C coupling in hiPSC-CMs, we performed confocal laser scanning microscopy in conjunction with the Ca^2+^ fluorescent indicator, fluo-4, to determine the relationship between occurrence of spontaneous Ca^2+^ sparks and activation of L-type Ca^2+^ channels. The dynamic parameters of Ca^2+^ spark unveiled novel fundamental characteristics of Ca^2+^ handling and regulation in hiPSC-CMs.

## Materials and Methods

### Generation of hiPSC Lines

Human induced pluripotent stem cells (hiPSCs) were generated in our laboratory from human neonatal dermal fibroblasts (ATCC, USA) via retroviral-based ectopic expression of Yamanaka factors (Oct-4, Sox-2, c-Myc and Klf-4) [6]. The pluripotent status of hiPSCs was confirmed by their expression of pluripotent markers and by their pluripotent differentiation potential including embryoid body (EB) formation and cardiac differentiation *in vitro* and teratoma formation *in vivo*. hiPSCs were maintained on mouse embryonic fibroblasts (MEF) feeder in hESC medium (80% Knockout Dulbecco's Modified Eagle Medium or DMEM, 20% Serum replacement, 1% non-essential amino acid, 1 mM L-glutamine, 0.1 mM beta-mercaptoethanol and 4 ng/mL bFGF). Unless specified, all culture reagents were from Invitrogen.

### Cardiomyogenic Differentiation of hiPSCs

Embryoid bodies (EBs) were generated after mechanical dissection of hiPSCs and maintained in suspension culture in a cardiomyogenic medium or CARM which contains DMEM High Glucose 485 mL, L-Glutamine 5 mL, NEAA 5 ml, Selenium Transferrin 5 mL (Sigma) and 2-mercaptoethanol 3.5 µL supplemented with 5 µM SB 203580 (Sigma), a specific p38-MAPK inhibitor [10] in low adherent 6-well plates. Subsequently, EB aggregates were formed and contracting outgrowths emerged from Day 12 onwards. After Day 15, the contracting EB aggregates were plated on 0.1% gelatin in DMEM containing 2% FBS. On Day 21, the contracting area of EBs were mechanically dissected out and enzymatically dissociated in Collagenase B (Roche) to small cell clusters containing 15∼30 cells according to published protocol with some modifications [11]. Dissociated CMs were continually cultured in DMEM+2% FBS for 1 week before testing. All tested hiPSC-CMs were kept at the same time point (6-weeks) post the initiation of cardiac differentiation.

### Immunocytochemistry

Detail description of immunocytochemistry assay of hiPSCs is described in Text S1. Briefly, immunocytochemistry assay of hiPSC-CMs was performed with following procedure. Dissociated cultured on glass coverslips were fixed using 4% paraformaldehyde and permeabilized with 0.1% Triton-X-100 (Sigma). After blocking with 5% goat serum in PBS for 1 h at room temperature, cells stained with mouse anti-human cardiac sarcomeric alpha-actinin (α-actinin) (clone EA-35, Sigma) and mouse anti-human cardiac myosin heave chain, beta (β-MHC) (Alexis Biochemicals, FL, USA). Next, the primary mAbs was removed and replaced with goat anti–mouse IgG (A11001 Alexa Fluor 488, Invitrogen) for 1 hour. Nuclei were counter stained with DAPI.

### Recording of Action Potential

Dissociated hiPSC-CMs were cultured on 3.5 cm glass-bottom dishes (WillCo-dish® Glass Bottom Dishes, the Netherlands). The spontaneous action potentials (AP) were recorded from hiPSC-CMs in normal Tyrode’s solution ((in mM): NaCl 140, KCl 5.4, CaCl_2_ 1.8, MgCl_2_ 1, glucose 10, HEPES 10, pH 7.40 with NaOH) under current-clamp conditions by an Axopatch 200B patch clamp amplifer (Axon instrument, USA). Patch pipettes, with a resistance of 2–4 MΩ, were pulled by a horizontal puller (Model P-97, Sutter Instrument, USA), and filled with an internal solution containing (in mM): KCl 130, MgCl_2_ 1, MgATP 3, EGTA 10, and HEPES 10, pH 7.20 with KOH. In addition, the effect of isoproterenol (ISO, 1 µM) was tested on ventricular-like hiPSC-CMs.

### Confocal Ca^2+^ Imaging

Ca^2+^ transients and Ca^2+^ sparks were recorded in ventricular-like hiPSC-CMs using a LSM-710 laser scanning confocal microscope (Carl Zeiss, Inc Germany) with a 40×, 1.3 numerical aperture oil immersion objective and axial resolutions of 1.5 µm [12]. Briefly, cells were loaded with 6 µg/mL Fluo-4 AM for 15 min at 37°C, and than were placed in the experimental chamber containing normal Tyrode’s solution. Fluo-4 was excited at 488 nm, and fluorescence emission was measured at >505 nm. Images were acquired in the line-scan (X-T) mode with 512 pixels (pixel intervals of 0.15 µm) per line at a rate of 3 ms per scan. Two-dimensional images were obtained with the confocal microscope operating in the frame-scan (X-Y, 512×512 pixels) mode. SR Ca^2+^ load was estimated by rapid application of 10 mM caffeine with a multi-channel rapid application system.

The intracellular free Ca^2+^ ([Ca^2+^]_i_) concentration was determined at the end of Ca^2+^ imaging experiment by the ionomycin method (Text S1 and Figure S1).

### Statistical Analysis

Ca^2+^ sparks, Ca^2+^ transients, and SR Ca^2+^ contents were analyzed using a computer program written in IDL 5.4 software, as previously described [13]. Results were expressed as mean ± standard error of the mean (SEM). Statistical significance was determined using Student-*t* test or non-parametric Kruskal-Wallis test, when appropriate. A *p* value <0.05 was considered to be statistically significant.

## Results

### Characterization of hiPSCs

**Figure 1:**
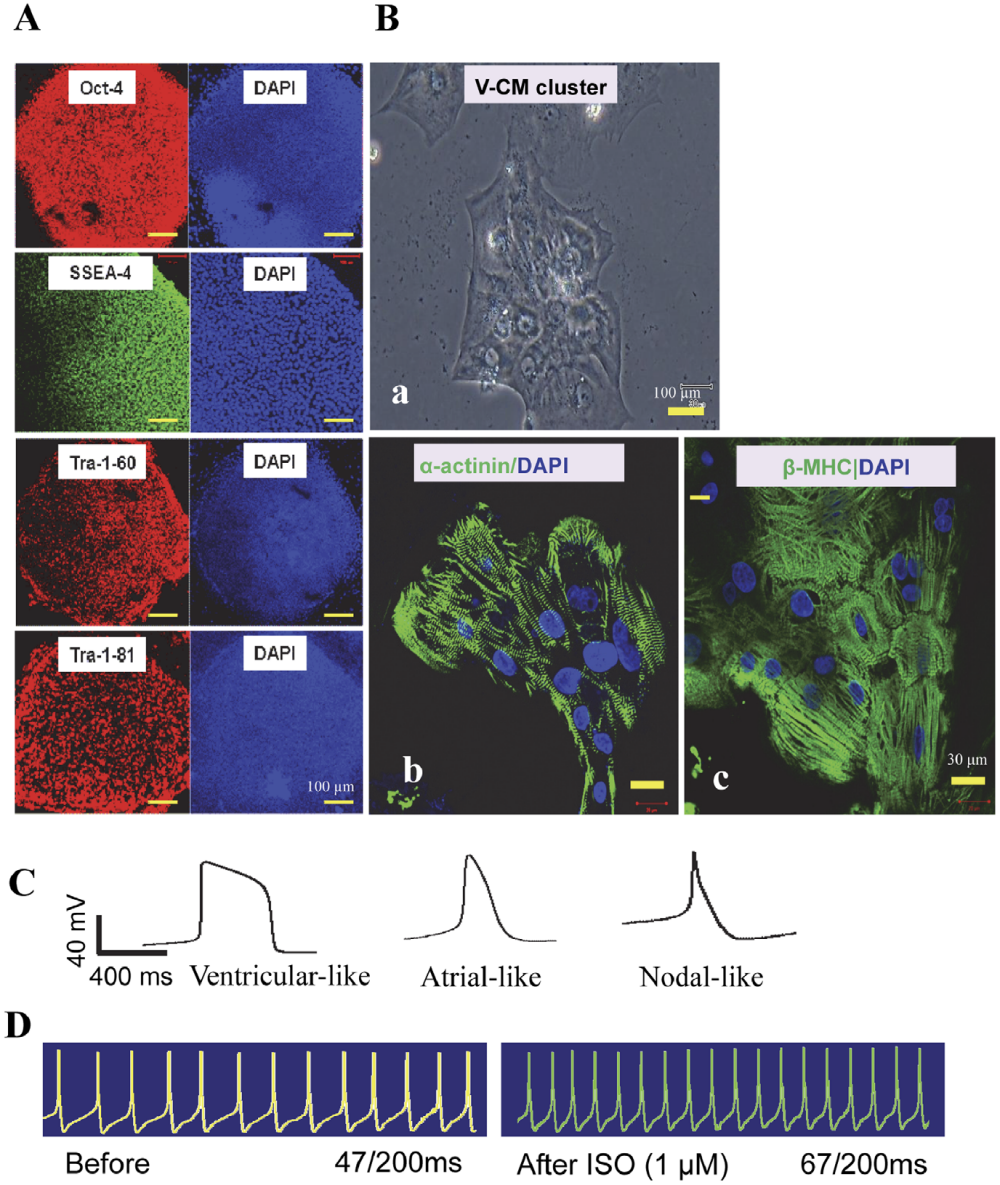
Characterization of hiPSCs and hiPSC-derived CMs. (A) Immunofluorescent staining of hiPSC colonies with antibodies against Oct-4, SSEA-4, TRA-1-60 and TRA-1-81. (B) The hiPSC-CMs differentia4ed from above hiPSC line. (Ba) The phase-contrast light micrograph images of a V-CM cluster. (Bb and Bc) Immunofluorescent staining hiPSC-CMs with antibodies against alpha-actinin and beta-MHC, respectively. Nuclei were stained with DAPI. (C) Action potential traces of ventricular-, atrial- and nodal-like CMs derived from hiPSCs. (D) Response of a ventricular-like hiPSC-CM to ISO recorded with patch-clamp. Abbreviations: ISO, isoproterenol.

hiPSC lines showed characteristic hESC-like morphology and comparable expression of hESC pluripotent markers including Oct-4, SSEA-4, TRA-1-81 and TRA-1-60 (Figure 1A). They formed teratoma in SCID mice and maintained normal karyotypes (data not shown).

### Cardiomyocyte Differentiation of hiPSCs

Under cardiac differentiation condition, spontaneously contracting EBs were derived from hiPSC lines after 15 days. Dissociated hiPSC-CMs in the small clusters containing 15∼30 CMs with uniformed subtypes (Figure 1Ba), were found to express sarcomeric alpha-actinin (α-actinin) and beta-myosin heavy chain (β-MHC) with cross striations that were typical of CMs derived from hESCs (Figure 1Bb, c). Moreover, three subtypes of CMs were identified including ventricular-, atrial- and nodal-like CMs (V-CMs, A-CMs and N-CMs) were identified in hiPSC-CMs (Figure 1C). The subtypes of hiPSC-CMs were determined by their typical AP properties including, action potential amplitude (APA), action potential duration (APD) and dV/dtmax. From a total of 100 cardiomyocytes examined, the percentages of V-CMs, A-CMs and N-CMs were about 61%, 17.4% and 21.6%, respectively (Table S1). It was noted that small clusters of cardiomyocytes (15∼30 cells) dissociated from contracting EBs tended to contain exclusively homogenous subtypes of V-CMs and N-CMs (See Text S1). Furthermore, hiPSC-derived V-CMs (n = 5) showed a classical response towards ISO at minimal effective dose of 1 µM that induced contractions per 100ms at baseline and post ISO treatment at 26.4±5.2 and 35.2±6.4 (p<0.001) respectively (Figure 1D). However, atrial-and nodal-like CMs were not tested due to low yield of such subtypes in the hiPSC-CM preparation. Collectively, our data confirmed that hiPSC-CMs displayed cardiac structures and physiological function of cardiomyocytes similar to those of hESC-CMs.

### Spontaneous Ca^2+^ Transients in hiPSC-CMs

**Figure 2:**
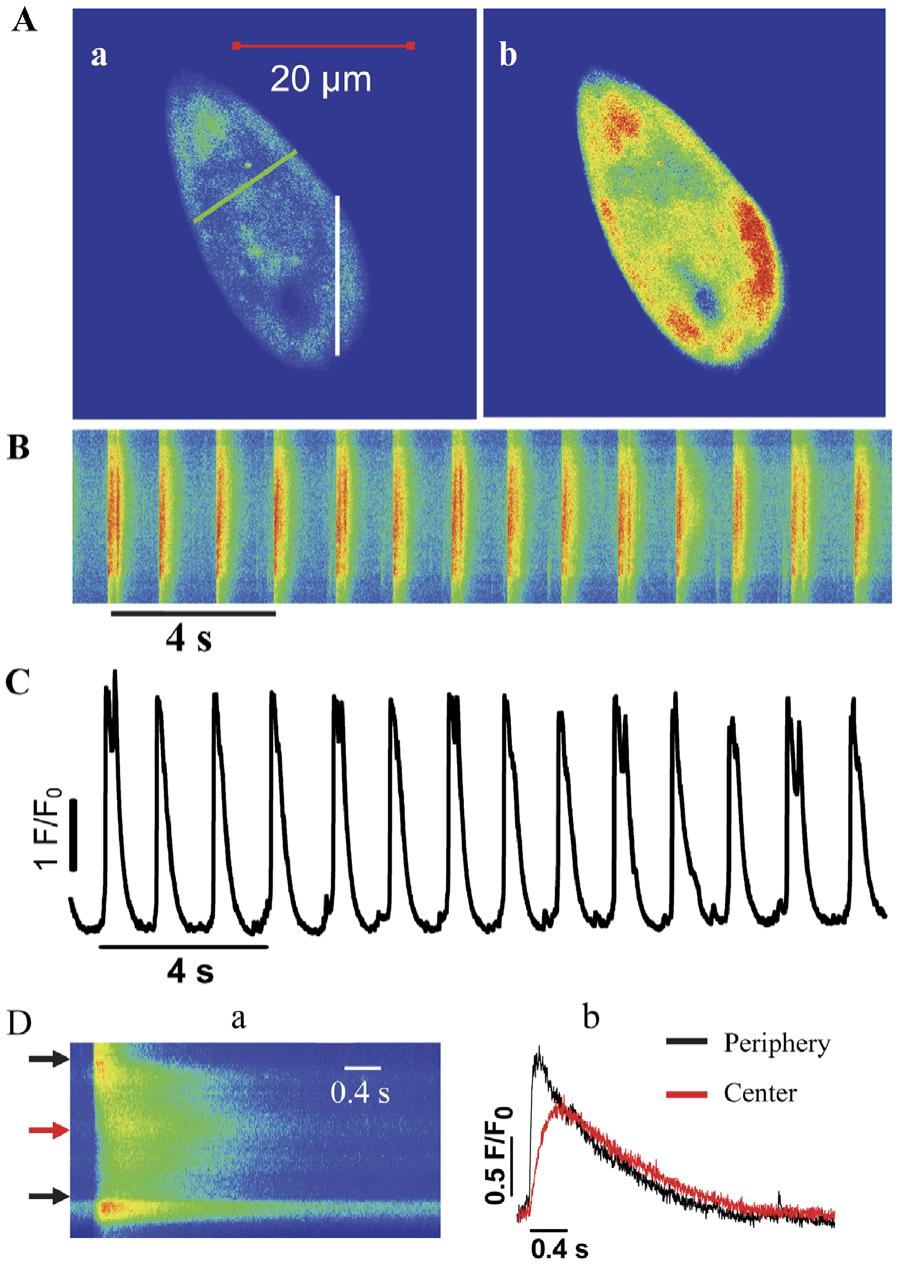
Spontaneous Ca^2+^ transients in hiPSC-CMs. (A) Representative frame-scan (X-Y mode) images of spontaneous Ca^2+^ transients (a and b). (B) A typical line scan (X-T mode) image of spontaneous Ca^2+^ transients obtained from white line in panel Aa and (C) the corresponding amplitudes (F/F_0_) of Ca^2+^ transients (n = 16). (D) A representative transverse line scan (X-T mode) image obtained from green line in panel Aa (a) and the corresponding intensity profiles (b) of Ca^2+^ transients. Abbreviations: F/F_0_, fluorescence (F) normalized to baseline fluorescence (F_0_); s, seconds.

Figure 2Ab shows representative Ca^2+^ transients obtained from sequential images recorded by a frame-scan mode in single hiPSC-CM. A typical line-scan image of Ca^2+^ transient and its average fluorescence intensity were shown in Figure 2B and 2C. The average peak amplitude of Ca^2+^ transients (F/F_0_) was 3.8±0.7 in hiPSC-CMs. To observe spread patterns of Ca^2+^ transients of hiPSC-CMs, transverse line-scan images of Ca^2+^ transient were performed. As shown in Figure 2Da, Ca^2+^ increased first at the periphery of the cell before propagating towards the centre of the cell with a mean time delay of 46±15 ms (n = 7) (Figure 2Db). Calibration of [Ca^2+^]_i_ was performed as described in Text S1 and Figure S1.

In contrast to hiPSC-CMs, field stimulation evoked a rapid and uniform increase in intracellular Ca^2+^, and then Ca^2+^ quickly dropped homogeneously to resting levels in adult rat cardiomyocytes (n_rat_ = 5, n_cell_ = 12). The average amplitude of Ca^2+^ transients (F/F_0_) was 3.5±0.6 (Figure S2).

### Spontaneous Ca^2+^ Sparks in hiPSC-CMs

**Figure 3:**
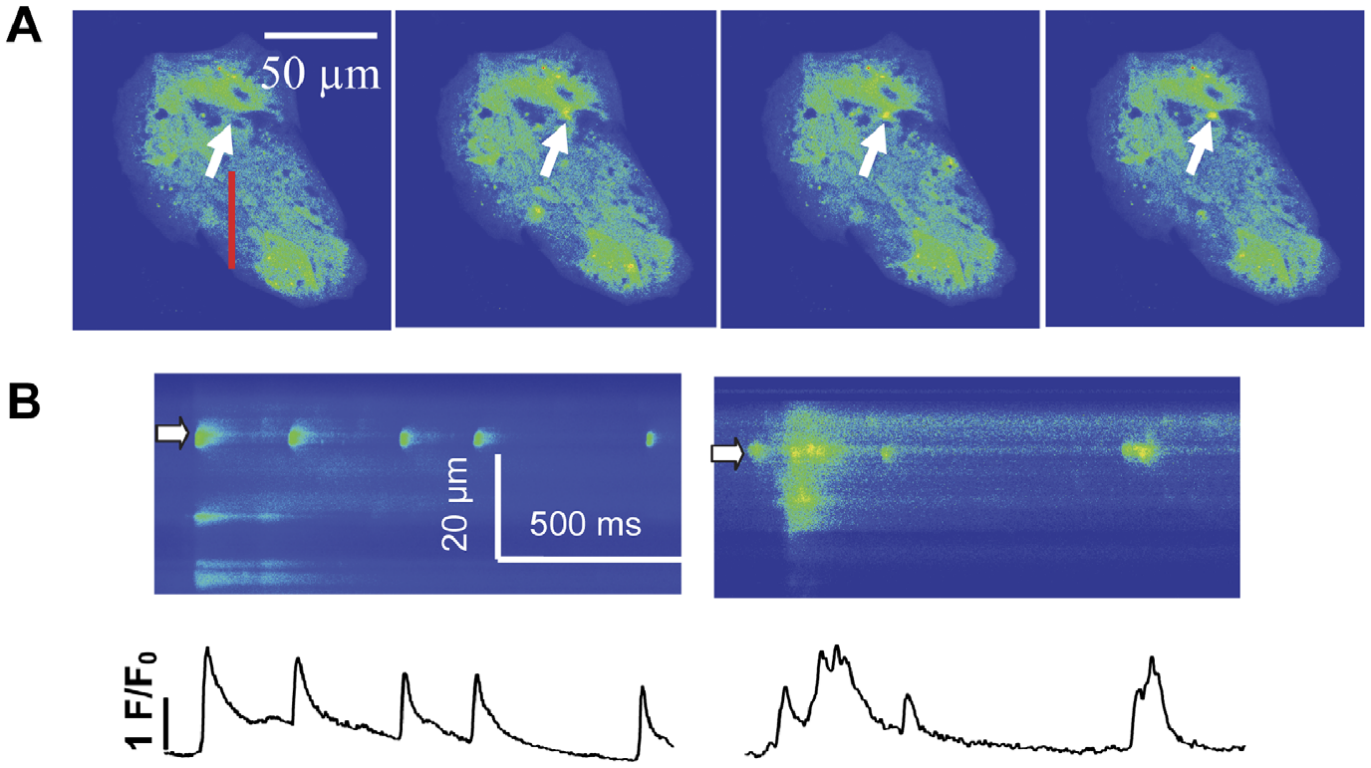
Spontaneous Ca^2+^ sparks in hiPSC-CMs. (A) Confocal images of spontaneous sparks were recorded in X-Y scanning mode. Representative spontaneous Ca^2+^ sparks occurred at the same site. (B) Two representative line scan (X-T mode) images of Ca^2+^ sparks obtained from red line in panel A at different times (top) and the intensity-time profiles of Ca^2+^ sparks at sites indicated by white arrows (bottom). Abbreviations: F/F_0_, fluorescence (F) normalized to baseline fluorescence (F_0_).

As shown in Figure 3A, serial frame-scan images on the same location of hiPSC-CMs showed a spontaneous elevation of local Ca^2+^ or Ca^2+^ sparks occurred inside the cytoplasm (arrow) at different times. To better characterize the spatial and temporal properties of Ca^2+^ sparks, line-scan imaging was carried out to monitor Ca^2+^ dynamics at 3 ms resolution in hiPSC-CMs. Fluorescence (the ratio of fluorescence to background fluorescence (F/F_0_)) profiles of Ca^2+^ sparks (bottom) were shown in Figure 3B. The repetitive Ca^2+^ sparks shown in Figure 3B indicated that individual sites could be repeatedly activated to generate Ca^2+^ sparks, even during the occurrence of spontaneous Ca^2+^ transients. In adult rat cardiomyocytes, repetitive Ca^2+^ sparks were seldom observed (<0.5% in present experiment, n_rat_ = 5, n_cell_ = 31) (Figure S3).

### Unique Characteristics of Spontaneous Ca^2+^ Sparks in hiPSC-CMs

**Figure 4:**
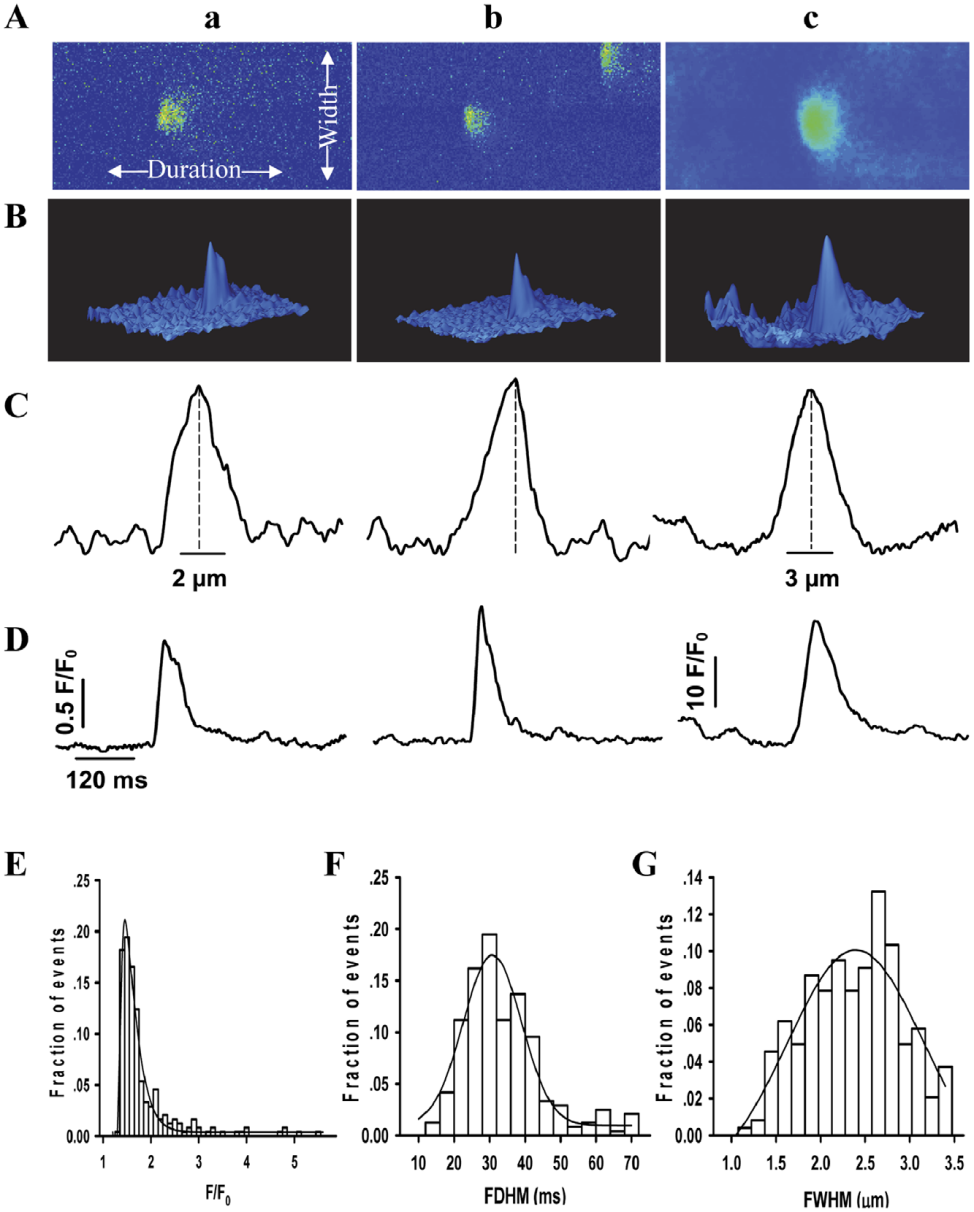
The characteristics of spontaneous Ca^2+^ sparks in hiPSC-CMs. (A) Two representative Ca^2+^ sparks (a and b) and an overlay of 160 original Ca^2+^ sparks (c) were obtained from the line-scan (X-T) images. (B) The three-dimensional surface plot of the Ca^2+^ spark in panel A. (C) The spatial width of Ca^2+^ sparks. (D) The duration of Ca^2+^ sparks. (E–G) show the distributions of Ca^2+^ sparks for F/F_0_, FDHM and FWHM, respectively. n_cell_ = 17, n_spark_ = 325. *P<0.05 vs. control. Abbreviations: F/F_0_, fluorescence (F) normalized to baseline fluorescence (F_0_); FDHM, full duration at half maximum; FWHM, full width at half maximum.

Figure 4Aa, b shows two typical line-scan images of Ca^2+^ sparks. An overlay of 160 original Ca^2+^ sparks was shown in Figure 4Ac. The spatial widths of Ca^2+^ sparks (Figure 4Ca,b) show that Ca^2+^ diffusion from the center of Ca^2+^ sparks to periphery was asymmetric, indicating that the distribution of RyRs in a cluster of Ca^2+^ release channels is anomalous or inhomogeneous in hiPSC-CMs. Ca^2+^ sparks also present multiple ridges in the three-dimensional plots (Figure 4Ba,b) and temporal profiles (Figure 4Da,b) of Ca^2+^ sparks, suggesting the these Ca^2+^ sparks may originate from one or several different clusters of RyRs. About 90% of Ca^2+^ sparks possess this temporal-spatial feature. However, the spatial width in an overlay of Ca^2+^ spark showed a symmetrical profile (Figure 4Cc).

In order to further determine the characteristics of Ca^2+^ sparks, we analyzed the amplitude (F/F_0_), spatial size (FWHM: full width at half maximum) or duration (FDHM: full duration at half maximum) of spontaneous Ca^2+^ sparks. Figure 4E–G showed the histogram for F/F_0_, FDHM and FWHM of Ca^2+^ sparks which we deduced the relationship between the amplitude and size distributions of Ca^2+^ sparks and the population of Ca^2+^ sparks from their histogram plots. The mean values for F/F_0_, FWHM and FDHM were 1.64±0.04, 2.31±0.03 µm and 30.9±0.6 ms, respectively. Ca^2+^ sparks between hiPSC-CMs and adult ventricular myocytes (n_spark_ = 302) have similar characteristics of Ca^2+^ sparks (Table S2).

### L-type Ca^2+^ Channels Contributes to Spontaneous Ca^2+^ Sparks and Ca^2+^ Transients

**Figure 5:**
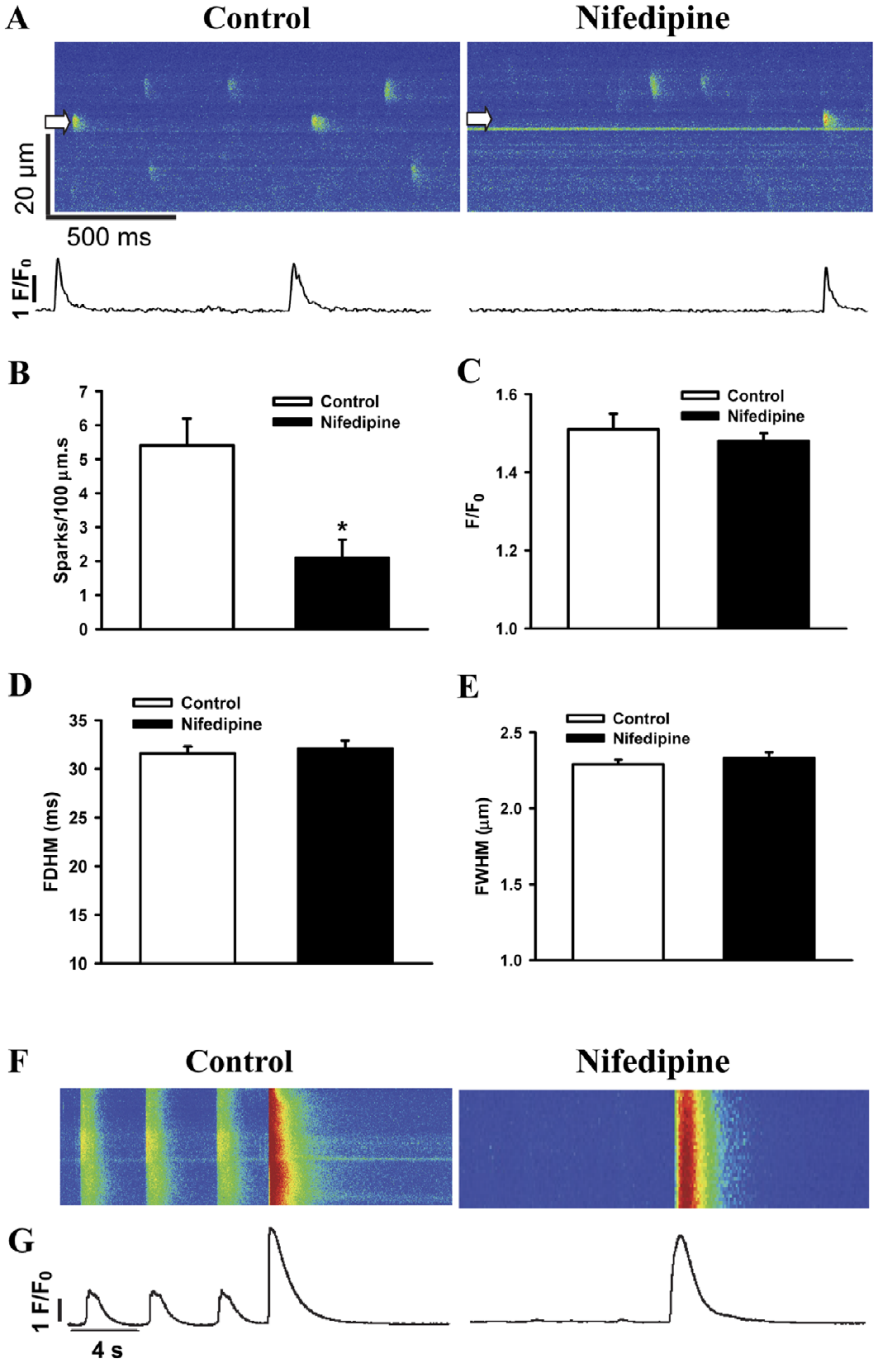
Effects of nifedipine on spontaneous Ca^2+^ sparks and SR Ca^2+^ loads in hiPSC-CMs. (A) Representative line scan (X-T) images of spontaneous Ca^2+^ sparks (top) and intensity-time profiles of typical sparks at positions denoted by white arrows (bottom) before and after the application of nifedipine. The average values of Ca^2+^ sparks for frequency (B), F/F_0_ (C), FDHM (D) and FWHM (E) before (n_spark_ = 213) and after (n_spark_ = 128) addition of nifedipine. (F) The line-scan images of caffeine-induced Ca^2+^ transients and (G) the corresponding F/F_0_ profiles before and after the application of nifedipine. n_cell_ = 12. *P<0.05 vs. control. Abbreviations: F/F_0_, fluorescence (F) normalized to baseline fluorescence (F_0_); FDHM, full duration at half maximum; FWHM, full width at half maximum; s, seconds.

To examine whether some of Ca^2+^ sparks were triggered by activation of RyRs associated with spontaneous L-type Ca^2+^ channel openings, effect of nifedipine (5 µM) on the rate of occurrence of spontaneous Ca^2+^ sparks was observed. As presented in Figure 5A and 5B, inhibition of L-type Ca^2+^ channels by nifedipine significantly reduced the frequency of occurrence of Ca^2+^ sparks without affecting F/F_0_, FDHM and FWHM of Ca^2+^ sparks (Figure 5C–E). Thus, nifedipine treatment had no significant effect on characteristics of individual Ca^2+^ sparks, indicating that nifedipine-sensitive and nifedipine-insensitive Ca^2+^ sparks are indistinguishable by virtue of their unitary properties. Additionally, nifedipine led to the complete elimination of Ca^2+^ transients in hiPSC-CMs (Figure S4). Therefore, Ca^2+^ influx via L-type Ca^2+^ channels contributes to whole-cell Ca^2+^ transients.

### L-type Ca^2+^ Channels Blockade did not Affect SR Ca^2+^ Load

SR Ca^2+^ load can directly affect Ca^2+^ transient amplitudes and Ca^2+^ spark characteristics. We therefore assessed effect of nifedipine on SR Ca^2+^ load in hiPSC-CMs. Figure 5F and 5G shows the line-scan images and amplitudes of Ca^2+^ transients elicited by the application of 10 mM caffeine under both control and in the presence of nifedipine. SR Ca^2+^ load was unaffected by nifedipine (4.9±0.5 in nifedipine vs 5.1±0.4 in control) which indicated that L-type Ca^2+^ channels blockade did not affect SR Ca^2+^ load in hiPSC-CMs.

### Effects of Extracellular Ca^2+^ Concentration on Ca^2+^ Sparks

**Figure 6:**
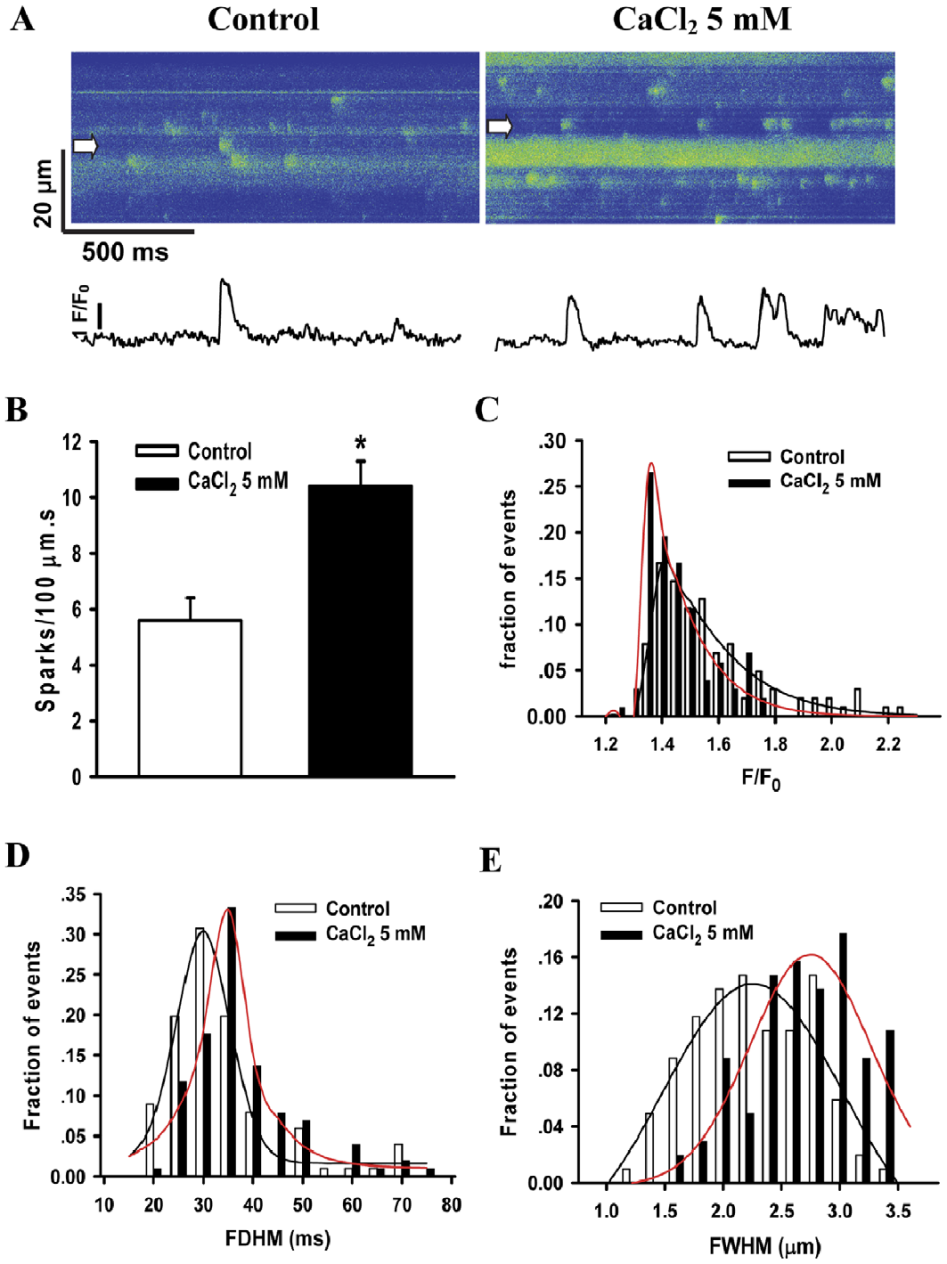
Effects of CaCl_2_ on spontaneous Ca^2+^ sparks in hiPSC-CMs. (A) Representative line-scan (X-T) images of spontaneous Ca^2+^ sparks (top) and the corresponding intensity-time profiles of typical sparks (bottom) before and after the application of 5 mM CaCl_2_. (B) The frequency of Ca^2+^ sparks. (C), (D) and (E) show the histograms for F/F_0_, FDHM and FWHM of Ca^2+^ sparks before (n_spark_ = 143) and after (n_spark_ = 318) application of 5 mM CaCl_2_, respectively. n_cell_ = 10. *P<0.05 vs. control. Abbreviations: F/F_0_, fluorescence (F) normalized to baseline fluorescence (F_0_); FDHM, full duration at half maximum; FWHM, full width at half maximum.

Ca^2+^ influx is an important trigger for SR Ca^2+^ release. To observe effect of extracellular Ca^2+^ concentration on Ca^2+^ sparks, 5 mM CaCl_2_ was applied in extracellular solution. Figure 6A shows the line-scan images of spontaneous Ca^2+^ sparks before and after the application of 5 mM CaCl_2_. It is clear that the frequency of Ca^2+^ sparks was 5.4±0.8 sparks/100 µm.s in control, significantly increased to 10.4±0.5 sparks/100 µm.s after application of 5 mM CaCl_2_ (Figure 6B). The histograms for FDHM and FWHM of Ca^2+^ sparks indicated an increase in big spark populations, the mean values for FDHM and FWHM were increased from 31.6±0.6 ms and 2.29±0.03 µm in control to 32.1±0.7 ms and 2.33±0.04 µm (All *P<0.05) in the presence of 5 mM CaCl_2_ (before n_spark_ = 143; after n_spark_ = 318; n_cell_ = 10), respectively (Figure 6D, E). However, the amplitude of Ca^2+^ sparks in the presence of 5 mM CaCl_2_ (1.48±0.02) was significantly lower than those in control (1.51±0.04) (*P<0.05) (Figure 6C). The results showed that elevated extracellular Ca^2+^ concentration resulted in an increase in big spark populations.

### Effects of Ryanodine on Ca^2+^ Sparks

**Figure 7:**
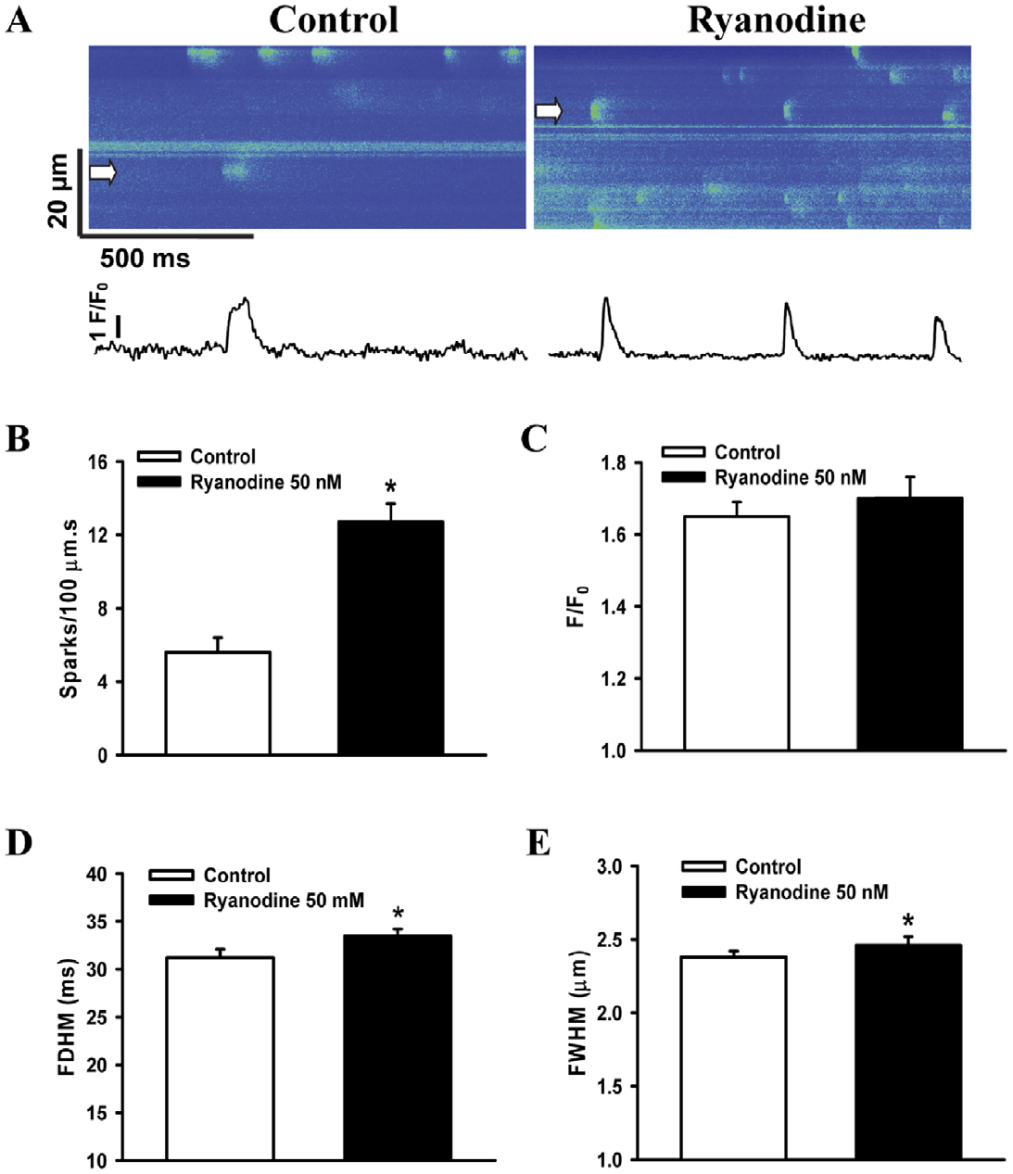
Effects of ryanodine on spontaneous Ca^2+^ sparks in hiPSC-CMs. (A) Representative line-scan (X-T) images of spontaneous Ca^2+^ sparks (top) and the corresponding intensity-time profiles of typical sparks (bottom) before and after the application of ryanodine. (B–E) show the mean values for frequency, F/F_0_, FDHM and FWHM of Ca^2+^ sparks before (n_spark_ = 163) and after (n_spark_ = 347) application of ryanodine, respectively. n_cell_ = 11. *P<0.05 vs. control. Abbreviations: F/F_0_, fluorescence (F) normalized to baseline fluorescence (F_0_); FDHM, full duration at half maximum; FWHM, full width at half maximum.

Ca^2+^ sparks are local and transient calcium release events from a cluster of RyRs in the SR. Delineating the properties of RyRs in hiPSC-CMs is thus a matter of fundamental importance to Ca^2+^ sparks. In the present study, the spark frequency FDHM and FWHM showed significant increase (P<0.05), whereas F/F_0_ was not significant changed after application of 50 nM ryanodine (before n_spark_ = 163; after n_spark_ = 347; n_cell_ = 11), when compared with control (Figure 7A–E). These results indicated that ryanodine could increase the size of Ca^2+^ sparks in hiPSC-CMs.

## Discussion

In adult cardiac myocytes, Ca^2+^ spark is an infrequent and stochastic elementary event of Ca^2+^ release [2]. Ca^2+^ sparks are often associated with the transverse tubules (TTs) at the Z-disk of a sarcomere where RyRs and L-type Ca^2+^ channels colocalize [12], [14], [15]. Furthermore, repetitive Ca^2+^ sparks may originate from the same RyR cluster [16]. In the present study, repetitive Ca^2+^ sparks emerged at the same sites were observed in hiPSC-CMs. In contrast, such phenomenon has rarely been reported in adult quiescent ventricular myocytes [16].

Similar to previous reports [17]–[19], the Ca^2+^ sparks in hiPSC-CMs were not associated with Ca^2+^ transients or Ca^2+^ wave propagation throughout the cells (Figure 3B). This phenomenon suggests that release units in those cells are separated by critical distances [20].

In adult mammalian ventricular myocytes, the T-tubules are the main site of E-C coupling and ensure spatially and temporally homogenous Ca^2+^ release throughout the cell [21]. Due to the lack of T-tubule, Ca^2+^ sparks are restricted to the cell periphery in neonatal cells and rabbit Purkinje cells whereby the SR membrane is associated directly with the plasma membrane which is similar to the dyadic cleft in ventricular myocytes [17]–[19], [22]. Studies with mouse and human ESC-derived CMs have revealed that T-tubules are either absent [23], [24], or less developed with poorly organized T-tubule system [25], [26]. Our present study of hiPSC-CMs show a U-shaped Ca^2+^ wavefront with rise of Ca^2+^ occurs initially at the cell periphery and then diffuses to the centre of the cell with an obvious delay. This indicates spatial separation between L-type Ca^2+^ channels and RyRs in ultrastructural organization which is likely due to the lack of t-tubules [7]. Similar findings of such segregation were reported previously [7], [18], [27], [28].

Spontaneous Ca^2+^ spark could be activated either by the cytosolic Ca^2+^ affecting the entire population of RyRs, or by Ca^2+^ entry into myocyte through the L-type channels. It is reported that about 50% of spontaneous sparks are attributable to spontaneous and infrequent openings of L-type Ca^2+^ channels at resting membrane potential [12]. The present results showed that blocking the L-type Ca^2+^ channels by nifedipine reduced over 60% of the rate of spark occurrence suggesting that Ca^2+^ sparks in hiPSC-CMs were triggered predominately by the L-type Ca^2+^ channel dependent triggering mechanisms.

Nifedipine did not inhibit caffeine-induced Ca^2+^ transients. Similar phenomena were also demonstrated after the application of nifedipine or Ca^2+^-free extracellular solution in cat ventricular myocytes or hiPSC-CMs [8], [29]. Accordingly, the main Ca^2+^ source for the caffeine-induced Ca^2+^ transients is not dominated by Ca^2+^ influx via L-type Ca^2+^ channels [8].

To study the effect of Ca^2+^ concentration on Ca^2+^ sparks, 5 mM CaCl_2_ was applied to extracellular solution. Under high extracellular Ca^2+^ condition, spontaneous Ca^2+^ sparks synchronously activated nearby Ca^2+^ release units and produced multiple sparks, called a “compound spark” or “macrospark”, from neighboring Ca^2+^ release units [30]. Similar observations were seen in Fig. 6, our findings were consistent with those reported in adult ventricular myocytes [31]. Therefore, Ca^2+^ sensitivity of RyRs in hiPSC-CMs was similar to those in adult ventricular myocytes. The decrease in the amplitude of sparks might be related to a decrease in the amount of Ca^2+^ release per RyR opening.

In the present study, ryanodine elevated the rate of spark occurrence and the temporal and spatial properties of Ca^2+^ sparks, without affecting the amplitude of Ca^2+^ sparks. RyR2 is the predominant RyR isoform in cardiac muscle, this RyR isoform is essential for E-C coupling and Ca^2+^ sparks in cardiac myocytes [4]. It was reported that Ca^2+^ sparks activity was absent after genetic ablation of RyR2 in stem cell-derived cardiomyocytes [4]. The expression of RyR2 gene in hiPSC-CMs has been confirmed previously [7], [8]. Therefore, our results indicated that a functional RyR2-mediated SR Ca^2+^ release is present in hiPSC-CMs.

In hiPSC/hESC-CMs, the mechanism of E-C coupling remains contentious. Some reports supported classical model of E-C coupling [8], [32]. Alternatively, it was suggested that Ca^2+^ used by the contractile machinery was provided by transsarcolemmal influx and not by SR Ca^2+^ release [33]. In the present study, the elimination of Ca^2+^ transients and the decrease of Ca^2+^ spark frequency in the presence of nifedipine demonstrated that Ca^2+^ transients in hiPSC-CMs were tightly regulated by the CICR mechanism during E-C coupling. To discount inter-line variations, hiPSC lines from additional 3 healthy subjects were examined and similar (no statistical difference) Ca^2+^ properties were observed among the cardiomyocytes (with same post cardiac differentiation time point) derived from all 4 lines, including the one presented in this study (Table S3). There was no significant difference in Ca^2+^ spark properties in hiPSC-CMs differentiated from different clones.

Electrophysiological property of pluripotent stem cell-derived CMs may vary due to culture duration of hiPSC-CMs [34]. In our study, cardiomyocytes maintained under culture conditions from 4 to 7 weeks post cardiac differentiation were compared in their characteristics of Ca^2+^ sparks and no significant differences were identified (data not shown). Nevertheless, long-term following up studies were not performed due to low yield of cardiac differentiation.

In summary, we identified spontaneous Ca^2+^ sparks and documented their fundamental characteristics in hiPSC-CMs. We found that the Ca^2+^ sparks in hiPSC-CMs share similar temporal and spatial properties with adult cardiomyocytes. Moreover, RyRs are functioning in hiPSC-CMs and a majority of spontaneous Ca^2+^ sparks is L-type Ca^2+^ channel dependent. However, the Ca^2+^ sparks in hiPSC-CMs appear to be stochastic with a tendency of repetitive occurrence at some sites. Such phenomenon might be attributed to a heterogeneous array of RyRs due to the lack of T tubules or immature T-tubule system in hiPSC-CMs.

## Supporting Information

Figure S1 **Measurement of [Ca^2+^]_i_ by using ionomycin.** (A) Representative line scan (X-T) image of Ca^2+^ transients before and after the application of ionomycin and EGTA. (B) The fluorescent intensity profiles of Ca^2+^ transients in A. (C) The Ca^2+^ concentrations of spontaneous Ca^2+^ transients were calculated by using equation: [Ca^2+^]I = K_d_[(F−F_min_)/(F_max_−F)]. Abbreviations: K_d_, the dissociation constant value of a fluorescence; F, the measured fluorescence value; F_max_, the fluorescence value with 2 µM ionomycin; F_min_, the fluorescence value with Ca^2+^-free bath solution containing 5 mM EGTA.
(TIFF)

Figure S2 **The characteristics of Ca^2+^ transients in rat cardiomyocytes.** A representative line-scan (X-T) image of Ca^2+^ transient recorded from field stimulated rat cardiomyocyte (top) and the corresponding intensity profiles (bottom) of Ca^2+^ transient. n_rat_ = 5, n_cell_ = 12. Abbreviations: F/F_0_, fluorescence (F) normalized to baseline fluorescence (F_0_).
(TIFF)

Figure S3 **The characteristics of spontaneous Ca^2+^ sparks in rat cardiomyocytes.** (A) A representative line-scan (X-T) image of Ca^2+^ sparks recorded from rat cardiomyocytes. (B) A typical Ca^2+^ spark from the cells indicated by arrow in A. (C) The three-dimensional surface plot of the Ca^2+^ spark in B. (D) The spatial width of Ca^2+^ spark. (E) The duration of Ca^2+^ spark. Abbreviations: F/F_0_, fluorescence (F) normalized to baseline fluorescence (F_0_).
(TIFF)

Figure S4 **Effects of 5 µM nifedipine on spontaneous Ca^2+^ transients in hiPSC-CMs.** Representative line scan (X-T) images (top) and the corresponding intensity profiles (bottom) of Ca^2+^ transients before and after the application ofnifedipine. n_rat_ = 5, n_cell_ = 13. Abbreviations: F/F_0_, fluorescence (F) normalized to baseline fluorescence (F_0_); s, seconds.
(TIFF)

Table S1 **The percentages of hiPSC-CM subtypes and the action potential properties.**
(DOCX)

Table S2 **Spatio-temporal properties of Ca^2+^ sparks in rat cardiomyocytes.**
(DOCX)

Table S3 **Characteristics of spontaneous Ca^2+^ sparks in hiPSC-CMs derived from additional hiPSC lines derived from 3 healthy subjects.**
(DOCX)

Text S1 **Materials and Methods S1, Results S1, Discussion S1, References S1.**
(DOCX)
