# Correction: Identification and Characterization of Calcium Sparks in Cardiomyocytes Derived from Human Induced Pluripotent Stem Cells

**DOI:** 10.1371/annotation/68ab355e-42c3-4843-8314-3a2ddbda02df

**Published:** 2013-09-13

**Authors:** Guang Qin Zhang, Heming Wei, Jun Lu, Philip Wong, Winston Shim

Due to an error, the original Figure 1A and Figure 1Ba were inadvertently mixed up with micrographs taken from other human induced pluripotent stem cell lines studied in our laboratory during the conduct of this reported study. The authors apologize for this error and are supplying a corrected Figure 1 that displays corrected micrographs.

Figure 1: 

**Figure:**
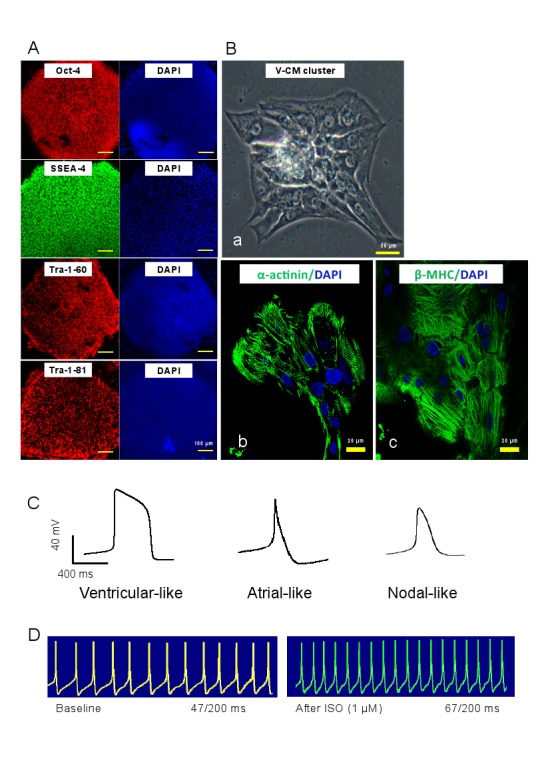


In addition, we would also like to address the following points:

- In the original figure, we mistakenly entered the value meant for Figure 1A (100 um) in Figure 1Ba and the value meant for Figure 1Ba (30 um) in Figure 1Bb and 1Bc. In the revised version, we have removed the multiple scale bars to avoid confusion and corrected those erroneous entries to reflect the true values.

- The figure legend should be corrected to read as below:

Figure 1. Characterization of hiPSCs and hiPSC-derived CMs. (A) Immunofluorescent staining of hiPSC colonies with antibodies against Oct-4, SSEA-4, TRA-1-60 and TRA-1-81. (B) The hiPSC-CMs differentiated from above hiPSC line. (Ba) The phase-contrast light micrograph images of a V-CM cluster. (Bb and Bc) Immunofluorescent staining of hiPSC-CMs with antibodies against alpha-actinin and beta-MHC, respectively. Nuclei were stained with DAPI. (C) Action potential traces of ventricular-, atrial- and nodal-like CMs derived from hiPSCs. (D) Response of a ventricular-like hiPSC-CM to ISO recorded with patch-clamp. Abbreviations: ISO, isoproterenol. 

