# Supplementary figures and images for: Identification and Characterization of Calcium Sparks in Cardiomyocytes Derived from Human Induced Pluripotent Stem Cells
Source: PLoS One. 2013 Feb 7;8(2):e55266. doi: 10.1371/journal.pone.0055266 (PMC3567046; doi:10.1371/journal.pone.0055266)

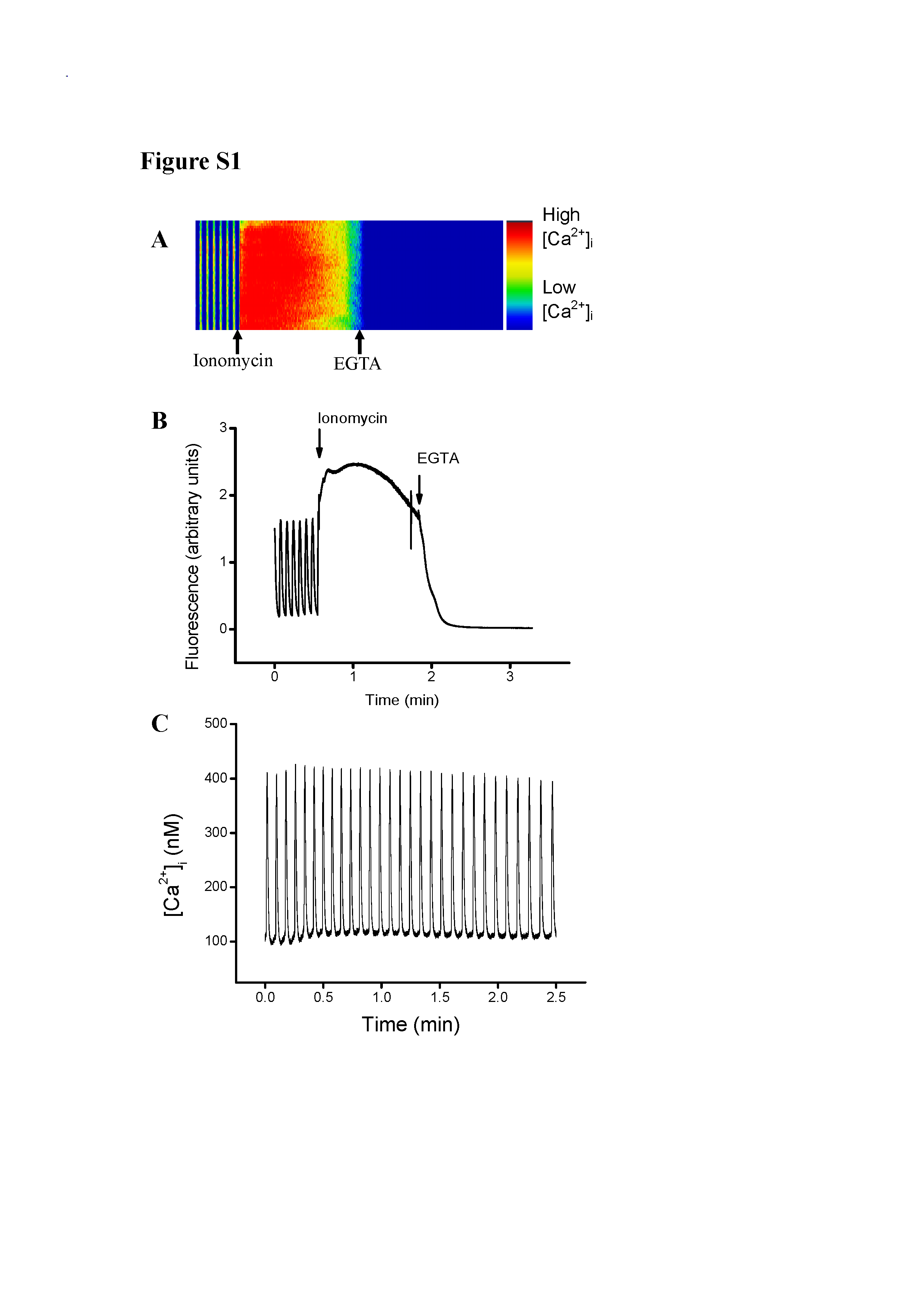

Supplement: Figure S1 — Measurement of [Ca2+]i by using ionomycin. (A) Representative line scan (X-T) image of Ca2+ transients before and after the application of ionomycin and EGTA. (B) The fluorescent intensity profiles of Ca2+ transients in A. (C) The Ca2+ concentrations of spontaneous Ca2+ transients were calculated by using equation: [Ca2+]I = Kd[(F−Fmin)/(Fmax−F)]. Abbreviations: Kd, the dissociation constant value of a fluorescence; F, the measured fluorescence value; Fmax, the fluorescence value with 2 µM ionomycin; Fmin, the fluorescence value with Ca2+-free bath solution containing 5 mM EGTA. (TIFF) [file pone.0055266.s001.tiff]

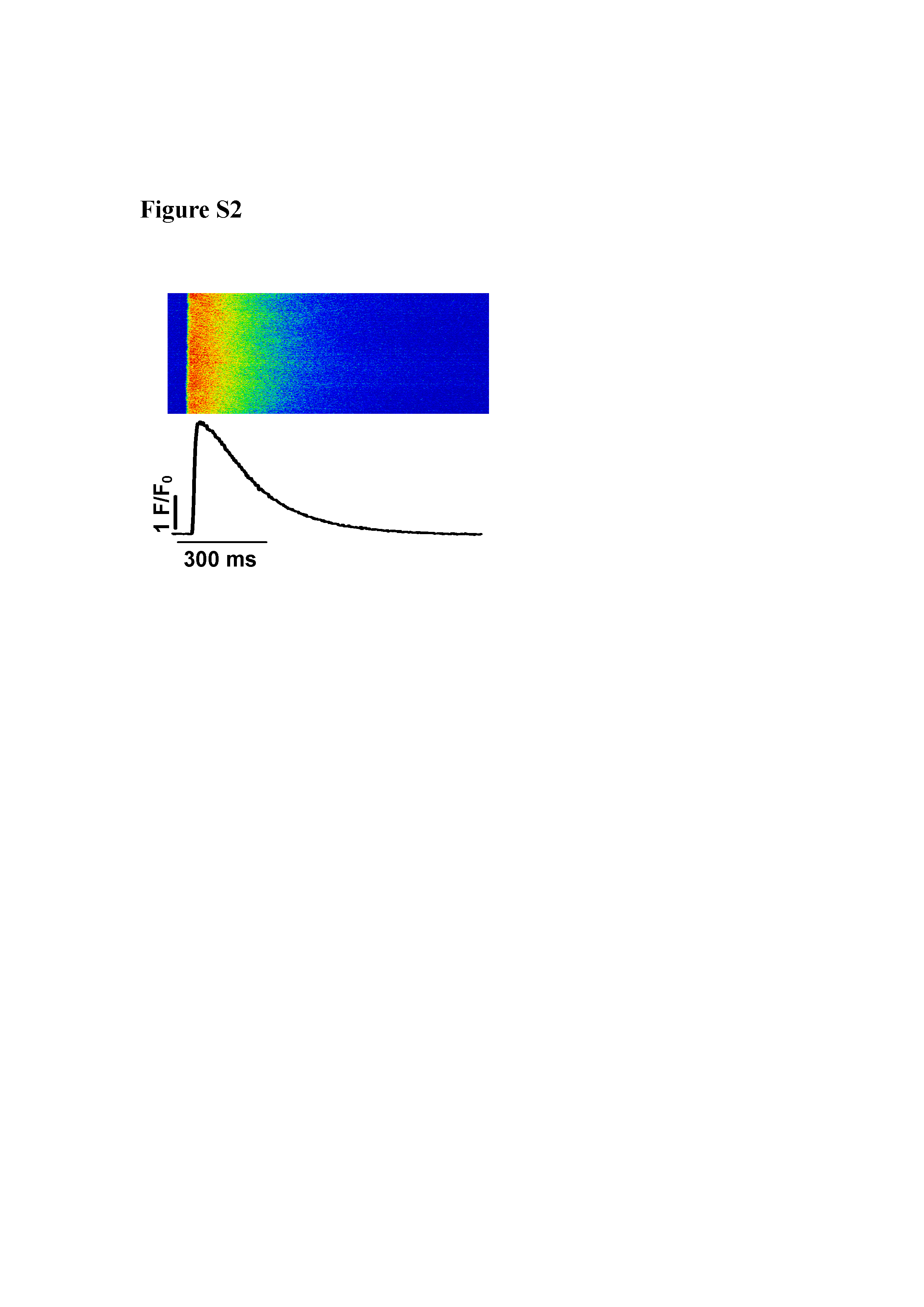

Supplement: Figure S2 — The characteristics of Ca2+ transients in rat cardiomyocytes. A representative line-scan (X-T) image of Ca2+ transient recorded from field stimulated rat cardiomyocyte (top) and the corresponding intensity profiles (bottom) of Ca2+ transient. nrat = 5, ncell = 12. Abbreviations: F/F0, fluorescence (F) normalized to baseline fluorescence (F0). (TIFF) [file pone.0055266.s002.tiff]

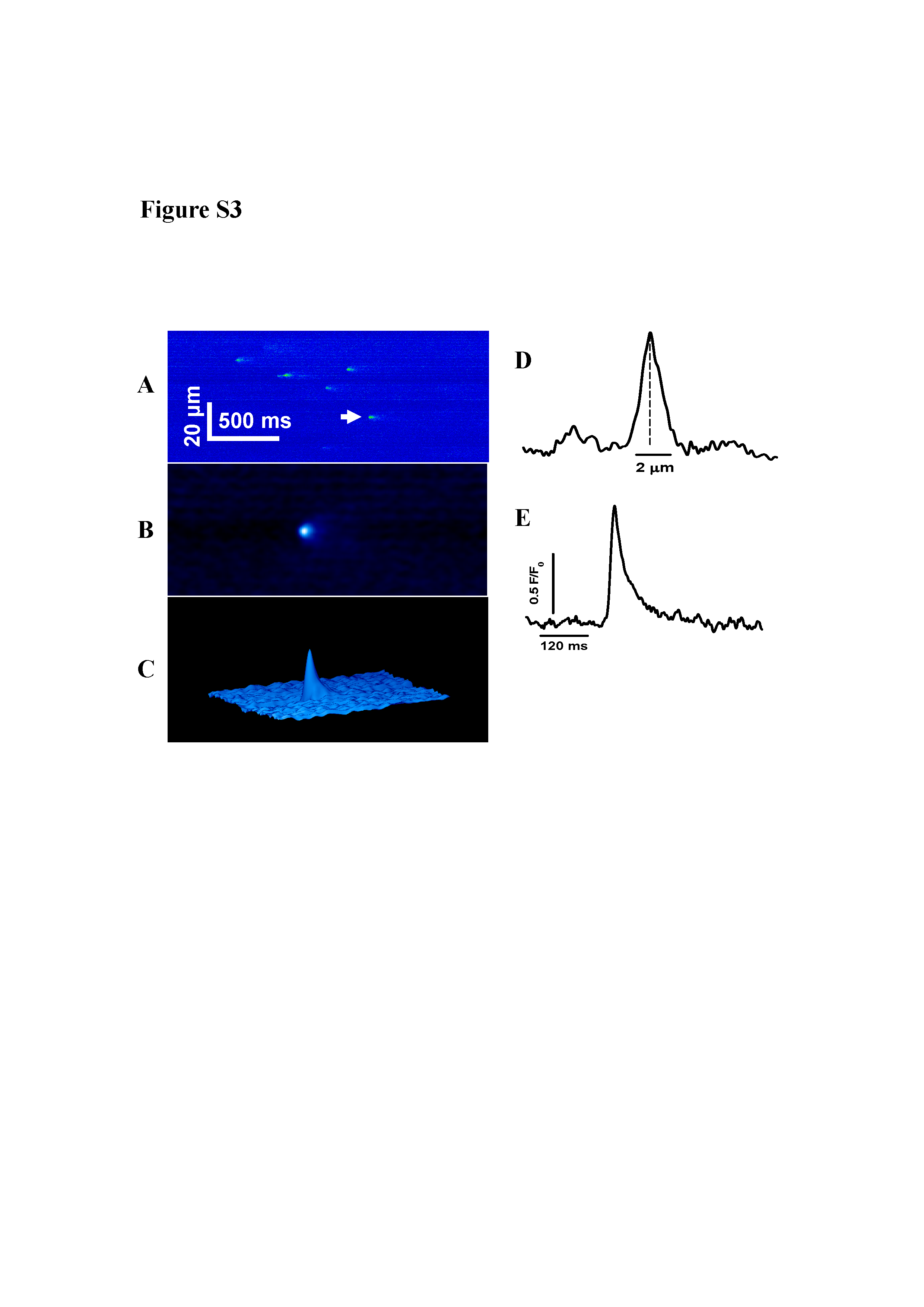

Supplement: Figure S3 — The characteristics of spontaneous Ca2+ sparks in rat cardiomyocytes. (A) A representative line-scan (X-T) image of Ca2+ sparks recorded from rat cardiomyocytes. (B) A typical Ca2+ spark from the cells indicated by arrow in A. (C) The three-dimensional surface plot of the Ca2+ spark in B. (D) The spatial width of Ca2+ spark. (E) The duration of Ca2+ spark. Abbreviations: F/F0, fluorescence (F) normalized to baseline fluorescence (F0). (TIFF) [file pone.0055266.s003.tiff]

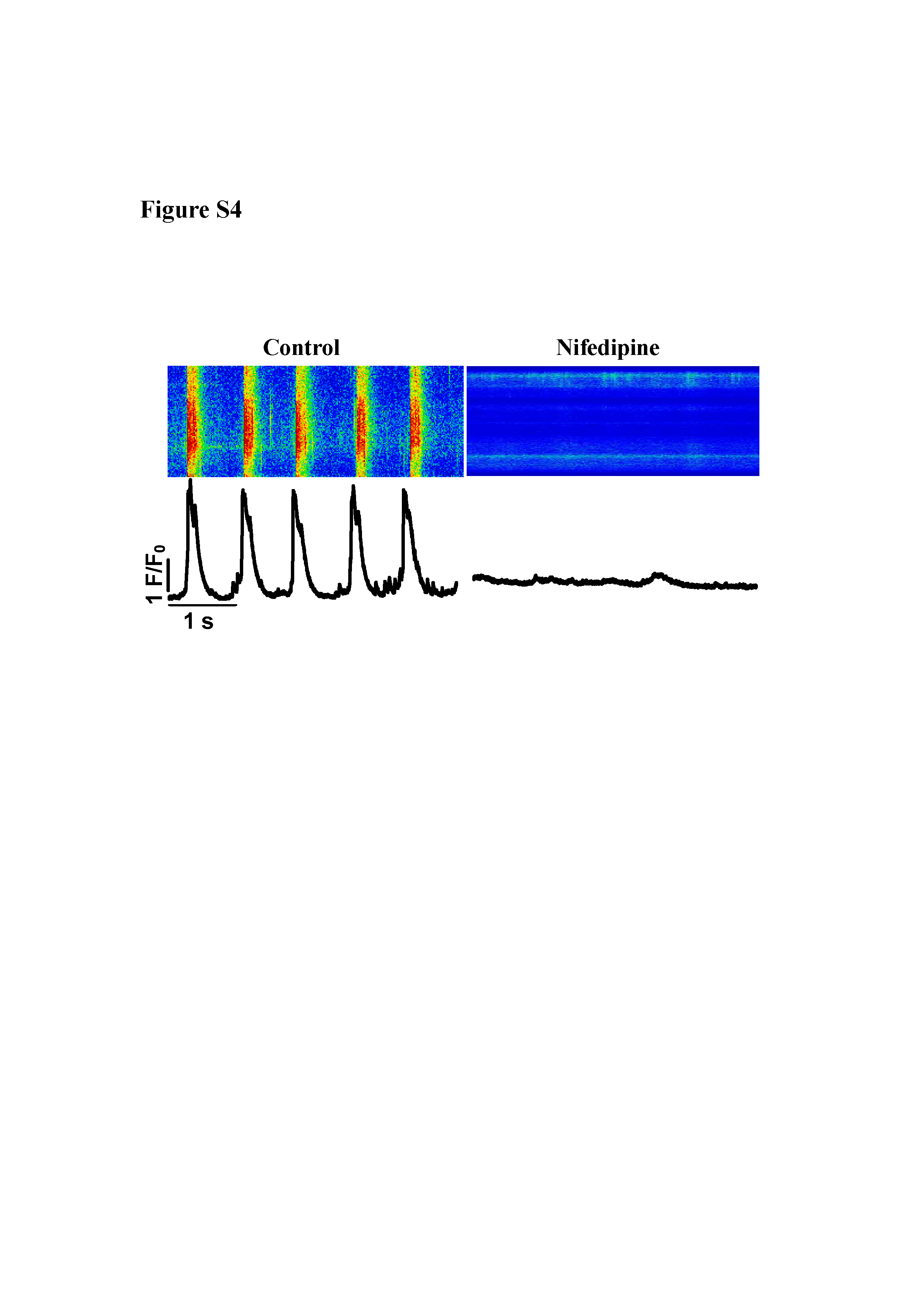

Supplement: Figure S4 — Effects of 5 µM nifedipine on spontaneous Ca2+ transients in hiPSC-CMs. Representative line scan (X-T) images (top) and the corresponding intensity profiles (bottom) of Ca2+ transients before and after the application ofnifedipine. nrat = 5, ncell = 13. Abbreviations: F/F0, fluorescence (F) normalized to baseline fluorescence (F0); s, seconds. (TIFF) [file pone.0055266.s004.tiff]
