# Supplementary material for: Identification and Characterization of Calcium Sparks in Cardiomyocytes Derived from Human Induced Pluripotent Stem Cells
Source: PLoS One. 2013 Feb 7;8(2):e55266. doi: 10.1371/journal.pone.0055266 (PMC3567046; doi:10.1371/journal.pone.0055266)
Supplement: Table S1 — The percentages of hiPSC-CM subtypes and the action potential properties. (DOCX) [file pone.0055266.s005.docx]

**Table S1.** The percentages of hiPSC-CM subtypes and the action potential properties.

| CM subtypes | Cells  n (% of total) | APA  (mV) | dV/dtmax (V/sec) | APD50  (ms) | APD90 (ms) |
| --- | --- | --- | --- | --- | --- |
| N-CMs | 23 (21.6%) | 61±2 | 7±2 | 146±31 | 212±31 |
| A-CMs | 20 (17.4%) | 79±3 | 15±3 | 175±29 | 263±52 |
| V-CMs | 67 (61.0%) | 81±9 | 16±3 | 393±73 | 479±26 |

Abbreviations: Ventricular- like CMs, V-CMs; Atrial-like CMs, A-CMs; Nodal-like CMs, N-CMs; APA, action potential amplitude; APD, action potential duration; dV/d*t*max, maximal rate of depolarization. Values given are mean ± SD.
