# Supplementary material for: Identification and Characterization of Calcium Sparks in Cardiomyocytes Derived from Human Induced Pluripotent Stem Cells
Source: PLoS One. 2013 Feb 7;8(2):e55266. doi: 10.1371/journal.pone.0055266 (PMC3567046; doi:10.1371/journal.pone.0055266)
Supplement: Table S2 — Spatio-temporal properties of Ca2+ sparks in rat cardiomyocytes. (DOCX) [file pone.0055266.s006.docx]

**Table S2. Spatio-temporal properties of Ca^2+^ sparks in rat cardiomyocytes.**

| n | Frequency  (Sparks/100 µm.s) | Amplitude (F/F_0_) | FDHM  (ms) | FWHM  (µm) |
| --- | --- | --- | --- | --- |
| 302 sparks | 4.6 ± 0.3 | 1.63 ± 0.04 | 26.1 ± 1.7 | 1.74 ± 0.12 |

Abbreviations: F/F_0_, fluorescence (F) normalized to baseline fluorescence (F_0_); FWHM, full width at half maximum; FDHM, full duration at half maximum. n_rat_ = 5 , n_cell_ = 31. Values given are mean ± SEM.
