# Supplementary material for: Identification and Characterization of Calcium Sparks in Cardiomyocytes Derived from Human Induced Pluripotent Stem Cells
Source: PLoS One. 2013 Feb 7;8(2):e55266. doi: 10.1371/journal.pone.0055266 (PMC3567046; doi:10.1371/journal.pone.0055266)
Supplement: Table S3 — Characteristics of spontaneous Ca2+ sparks in hiPSC-CMs derived from additional hiPSC lines derived from 3 healthy subjects. (DOCX) [file pone.0055266.s007.docx]

| hiPSC lines | Amplitude (F/F_0_) | FDHM (ms) | FWHM (µm) |
| --- | --- | --- | --- |
| Line 1 (121 Sparks) | 1.63 ± 0.04 | 32.1 ± 1.5 | 2.44 ± 0.12 |
| Line 2 (154 Sparks) | 1.48 ± 0.06 | 29.8 ± 0.9 | 2.17 ± 0.08 |
| Line 3 (130 Sparks) | 1.81 ± 0.06 | 35.2 ± 1.2 | 2.53 ± 0.07 |

**Table S3. Characteristics of spontaneous Ca^2+^ sparks in hiPSC-CMs derived from additional hiPSC lines derived from 3 healthy subjects.**

Abbreviations: F/F_0_, fluorescence (F) normalized to baseline fluorescence (F_0_); FWHM, full width at half maximum; FDHM, full duration at half maximum. Values given are mean ± SEM.
