# Supplementary material for: Identification and Characterization of Calcium Sparks in Cardiomyocytes Derived from Human Induced Pluripotent Stem Cells
Source: PLoS One. 2013 Feb 7;8(2):e55266. doi: 10.1371/journal.pone.0055266 (PMC3567046; doi:10.1371/journal.pone.0055266)
Supplement: Text S1 — Materials and Methods S1, Results S1, Discussion S1, References S1. (DOCX) [file pone.0055266.s008.docx]

SUPPORTING INFORMATION

**Identification and Characterization of Calcium Sparks in Cardiomyocytes Derived from Human Induced Pluripotent Stem Cells**

Guang Qin Zhang,^1,2┼^ Heming Wei,^1,3┼^ Jun Lu,^1^ Philip Wong,^1,3^ Winston Shim^1, 3*^

1 Research and Development Unit, National Heart Centre Singapore, Singapore, Republic of Singapore,

2 Department of Clinical Pharmacy, China Pharmaceutical University, Nanjing, China, 3 Cardiovascular & Metabolic Disorders Program, DUKE-NUS Graduate Medical School Singapore, Singapore, Republic of Singapore.

*The corresponding author: Tel.: +65 64350752, Fax: +65 62263972, E-mail: [winston.shim.s.n@nhcs.com.sg](mailto:winston.shim.s.n@nhcs.com.sg) (WS)

^┼^These authors contributed equally to this work.

**Materials and Methods S1**

Immunofluorescent staining of hiPSCs

Colonies of hiPSCs were fixed using 4% paraformaldehyde and permeabilized with 0.1% Triton-X-100 (Sigma). After blocking with 5% BSA for 1 h at room temperature, cells were stained with monoclonal antibodies anti-human Oct-4, SSEA-4, TRA 1–60, and TRA 1–81 (Millipore). Next, the primary mAbs was removed and replaced with goat anti–mouse IgG (A11001 Alexa Fluor 488 or A21422 Alexa Fluor 555, all from Invitrogen) for 1 hour).

Isolation of rat cardiomyocytes

Sprague-Dawley rats (~200 g) were obtained from the Experimental Animal Centre of National University of Singapore. All animals were kept in standard cages at 25 ± 1°C under a 12 h light/dark cycle. All animal experimental protocols were approved by the Institutional Anim!l Care and Use Committee (IACUC) of SingHealth, Singapore (No: 2011/SHS/676). All animals were performed under sodium pentobarbital anesthesia, and all efforts were made to minimize suffering.

Ventricular myocytes were isolated from rats by enzymatic digestion as previously described [1]. Finally, the cells were maintained in normal Tyrode solution in which the Ca^2+^ concentration was gradually adjusted to 1 mM.

Measurement of intracellular Ca^2+^ concentration [Ca^2+^]_i_

Calibration of [Ca^2+^]_i_ was performed as described previously [2]. The free [Ca^2+^]_i_ was then calculated by the equation: [Ca^2+^] = K_d_ [(F-F_min_)/(F_max_-F)]. F_max_ was obtained by adding 2 µM ionomycin until a`maximum fluorescence was obtained in the end of the experiment, then F_min_ was determined by adding 5 mM EGTA to the bathing solution until a minimum fluorescence was obtained. K_d_ is the dissociation constant value of a fluorescence, F is the measured fluorescence value, F_max_ is the maximum fluorescence value, F_min_ is the minimum fluorescence value.

**Results S1**

Identification of ventricular-, atrial- and nodal-like cardiomyocytes

hiPSC-CMs are known to contain ventricular-, atrial- and nodal-like subtypes of CMs. In this study, subtypes of hiPSC-CMs were determined by their characteristic action potential properties. The results of ‘patchable cells’ were summarized in Supplementary Table S1 (Identification of the subtypes of hiPSC-CMs by action potential properties).

Identification of small cell clusters hiPSC-CMs containing homogenous subtype of ventricular-like and nodal-like cardiomyocytes

It was repeatedly observed that small clusters of 15~30 hiPSC-CMs have a tendency of containing homogenous subtypes of nodal- or ventricular-like CMs. This was confirmed by the observation of 10 clusters that contained exclusively ventricular-like cardiomyocytes and 8 clusters that contained exclusively nodal cardiomyocytes (identify after Patch-Clamp analysis of over ~70% of “Patchable cells”).

Accordingly, a special strategy was developed to combine AP analysis with calcium imaging on the same subtype of cardiomyocytes. The dissociation procedure was modified to get small cell clusters contained 15-30 cells. Dissociated cardiomyocytes clusters were plated on glass-bottom dishes and subjected to Patch-Clamp assay first to measure action potential of 5~7 cells per cluster to identify the subtype of CMs. Next, intact cells within the labeled clusters were subjected to Ca^2+^ imaging assay. Due to the limited numbers of nodal clusters, Ca^2+^ spark assay was performed on cell clusters containing only ventricular-like hiPSC-CMs.

**Discussion S1**

Different subtypes of cardiomyocytes will feature different E-C coupling properties due to their structural difference such as T-tube developments in a cardiomyocytes. Previous studies have implicated that Ca^2+^ transients and Ca^2+^ sparks are mostly identified in ventricular cardiomyocytes as they have well developed T- tubes. Thus this study focused on ventricular-like cardiomyocytes.

We speculate that hiPSC-CMs could be originated from a few cardiac progenitor cells, generated at the early stage of cardiomyogenesis. Those cardiac progenitor cells could proliferate and give rise to a much larger number of cardiomyocytes [3]. Thus, a cluster (10~30) of hiPSC-CMs derived from an incompletely dissociated contraction EB could contain homogenous subtype of cardiomyocytes originated from a single cardiac progenitor cell. Such notion is supported by a latest study by Gupta and Poss who observed that the adult zebrafish heart originated from a few cell colonies can be tracked back to the early post fertilization stage [4].

**References S1**

1. Guangqin Z, Yu F, Dongmei Y, Xuemei H, Shuhua B, et al. (2004) Contribution of spontaneous L-type Ca^2+^ channel activation to the genesis of Ca^2+^ sparks in resting cardiac myocytes. Sci China C Life Sci 47: 31-37.
2. Chen JB, Tao R, Sun HY, Tse HF, Lau CP, Li GR. (2010) Multiple Ca^2+^ signaling pathways regulate intracellular Ca^2+^ activity in human cardiac fibroblasts. J Cell Physiol 223:68-75.
3. Yang L, Soonpaa MH, Adler ED, Roepke TK, Kattman SJ, et al. (2008). Human cardiovascular progenitor cells develop from a KDR+ embryonic-stem-cell-derived population. Nature 453: 524-528.
4. Gupta V, Poss KD. (2012) Clonally dominant cardiomyocytes direct heart morphogenesis. Nature 484: 479-484.
